# Discovery of a cytochrome P450 enzyme catalyzing the formation of spirooxindole alkaloid scaffold

**DOI:** 10.3389/fpls.2023.1125158

**Published:** 2023-02-03

**Authors:** Tuan-Anh M. Nguyen, Dagny Grzech, Khoa Chung, Zhicheng Xia, Trinh-Don Nguyen, Thu-Thuy T. Dang

**Affiliations:** ^1^ Department of Chemistry, Irving K. Barber Faculty of Science, University of British Columbia, Kelowna, BC, Canada; ^2^ Department of Natural Product Biosynthesis, Max Planck Institute for Chemical Ecology, Jena, Germany; ^3^ Chemistry Research Laboratory, University of Oxford, Oxford, United Kingdom; ^4^ Department of Chemistry, Faculty of Science, University of British Columbia, Vancouver, BC, Canada

**Keywords:** kratom, cytochrome P450, spirooxindole, CYP71, secoyohimbane

## Abstract

Spirooxindole alkaloids feature a unique scaffold of an oxindole ring sharing an atom with a heterocyclic moiety. These compounds display an extensive range of biological activities such as anticancer, antibiotics, and anti-hypertension. Despite their structural and functional significance, the establishment and rationale of the spirooxindole scaffold biosynthesis are yet to be elucidated. Herein, we report the discovery and characterization of a cytochrome P450 enzyme from kratom (*Mitragyna speciosa*) responsible for the formation of the spirooxindole alkaloids 3-*epi*-corynoxeine (3*R*, 7*R*) and isocorynoxeine (3*S*, 7*S*) from the corynanthe-type (3*R*)-secoyohimbane precursors. Expression of the newly discovered enzyme in *Saccharomyces cerevisiae* yeast allows for the efficient *in vivo* and *in vitro* production of spirooxindoles. This discovery highlights the versatility of plant cytochrome P450 enzymes in building unusual alkaloid scaffolds and opens a gateway to access the prestigious spirooxindole pharmacophore and its derivatives.

## 1 Introduction

**Figure 1:**
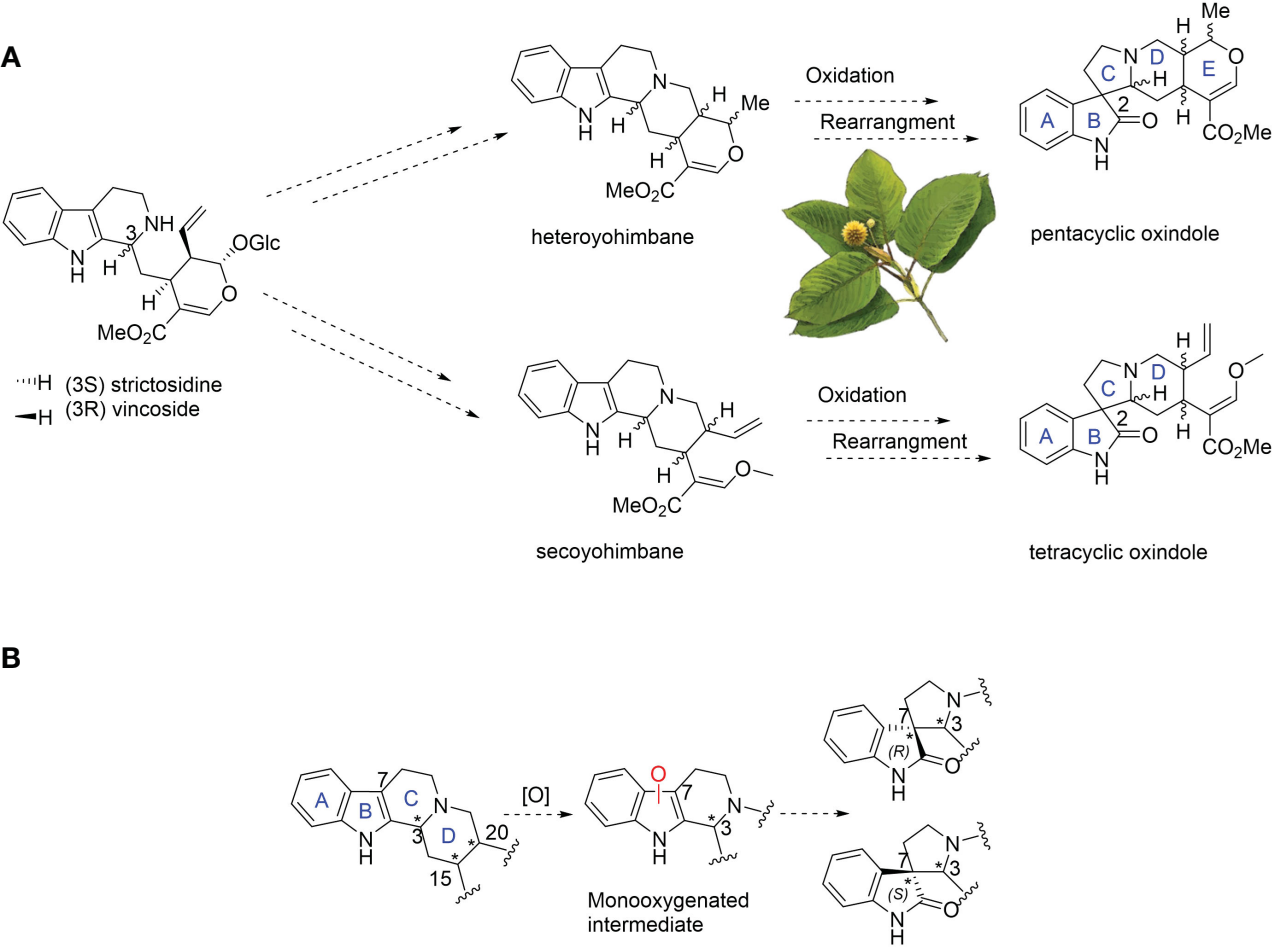
**(A)** Proposed biosynthesis of spirooxindole alkaloids from monoterpenoid indole alkaloid precursors in kratom (*Mitragyna speciosa*). The dotted arrows represent the unknown enzymatic steps. **(B)** Proposed oxidative rearrangement in the formation of spirooxindole alkaloids.

Spirooxindole alkaloids constitute a subclass of monoterpenoid indole alkaloids (MIAs) with a substituted carbonyl group at the C-2 position in the indole ring (**Figure 1A**). Since the first isolation of a spirooxindole alkaloid from the root of yellow jessamine (*Gelsemium sempervirens*) in 1870, many spirooxindole alkaloids have been reported from various plant genera, including *Mitragyna, Rauwolfia*, and *Vinca* (Bindra, 1973) (**Supplementary Figure 1**). The majority of spirooxindole alkaloids feature the unique spirooxindole scaffold in which the oxindole ring shares a single atom at the C-3 position with a cycloalkyl or a heterocyclic moiety (Zhou et al., 2020) derived from monoterpenoid indole alkaloids biosynthesis. As a valuable pharmacophore, spirooxindoles have recently attracted significant attention from chemists and biochemists for their diverse range of bioactivities. Examples include the tetracyclic corynoxeine and isocorynoxeine used in treating hypertension and stroke (Zhao et al., 2016), corynoxine and its isomer corynoxine B as potential agents to treat Parkinson’s disease (Chen et al., 2021), and mitraphylline with promising anti-tumour activity (**Supplementary Figure 1**) (García Giménez et al., 2010). Kratom (*Mitragyna speciosa*) and cat’s claw (*Uncaria rhynchophylla*) from the plant family Rubiaceae are well known for their spirooxindole alkaloid contents and thus have been the focus in studying spirooxindole biosynthesis. However, the low abundance of spirooxindole alkaloids in these plants (Manwill et al., 2022) makes biosynthetic elucidation a formidable task. Typically, spirooxindole alkaloids occur in pairs of interconvertible stereoisomers as the C-3–C-7 bond of the *p*-aminolactam group is prone to cleavage and reformation (**Figure 1B**) (Ahmad and Salim, 2015). The distinct stereogenic centers of the polycyclic scaffolds are the key feature contributing to the diversity of spirooxindole structures. Generally, the known spirooxindoles from *M. speciosa* are classified into two main structural types: secoyohimbane-type/tetracyclic and heteroyohimbane-type/pentacyclic structures (**Figure 1A**) (Bindra, 1973). While the underlying biochemistry is unknown, the spirooxindole group formation is speculated to be the result of an oxidative rearrangement at C-7 and C-3 positions in the tetrahydro-β-carboline moiety of the indole precursor in *M.* species (**Figure 1B**) (Shavel and Zinnes, 1962). Recent isotopic labelling studies supported that spirooxindole alkaloids could be generated in a one-step oxidative rearrangement from the tetrahydro-β-carboline moiety of seco- and hetero-yohimbine-type alkaloids such as corynantheine methyl ether and ajmalicine (Lopes et al., 2019). Among oxidative enzymes, cytochrome P450 enzymes (CYPs) are ubiquitous in plant specialized metabolism (Nguyen and Dang, 2021), especially members of the CYP71 family are key drivers of MIA diversification from simple seco- and hetero-yohimbine to various scaffolds, including sarpagan (Dang et al., 2018), strychnos (Tatsis et al., 2017; Hong et al., 2022; Wang et al., 2022), akuammilan (Wang et al., 2022), iboga (Farrow et al., 2019), and aspidosperma (Caputi et al., 2018; Qu et al., 2018). Therefore, we hypothesized that a CYP71 catalyzes the oxidative rearrangement of tetrahydro-β-carbolines to spirooxindoles. Using available *M. speciosa* transcriptome and genome (Brose et al., 2021) and OrthoFinder (Emms and Kelly, 2015), we identified and characterized a CYP71 enzyme that converts a secoyohimbine scaffold to a spirooxindole scaffold. This discovery opens a window into the largely unknown biosynthesis of spirooxindole alkaloids and offers a pioneering biocatalyst for sustainable synthetic routes of spirooxindoles from the tetrahydro-β-carboline scaffold.

## 2 Materials and methods

### 2.1 Identification, cloning of candidates and protein expression

Transcriptomes of spirooxindole alkaloids-producing plants (*M. speciosa*, *Rauwolfia serpentina*, and *G. sempervirens*), MIAs-producing but spirooxindole alkaloids-free plants (*Camptotheca acuminata*, *Amsonia hubrichtii*, *Cinchona ledgeriana*, *Nothapodytes nimmoniana*, *Ophiorrhiza pumila*, and *Catharanthus roseus*) and a MIAs-free plant (*Arabidopsis thaliana*) are publicly available from the Medicinal Plant Genomic Resources (http://mpgr.uga.edu/), the PhytoMetaSyn database (https://bioinformatics.tugraz.at/phytometasyn/), TAIR10 (https://www.arabidopsis.org/), and previous studies (Rather et al., 2018; Rai et al., 2021). TransDecoder was used to generate the proteomes, which were subsequently subjected to OrthoFinder for orthogroups analysis. Candidates belonging to the CYP71 family that act on the B ring of the indole moiety were selected to test for activities in the spirooxindole scaffold formation. We focused on the orthogroups containing CYP orthologues unique to *M. speciosa* (MsCYP). The phylogenetic tree of the MsCYP candidates with other reported CYPs involved in the biosynthesis of MIAs from different species was constructed by the Geneious Tree Builder program in the Geneious Prime software package (Biomatters). The amino acid sequence alignment of the MsCYP candidates was performed by the Geneious Prime software package (Biomatters). The open reading frames of MsCYP candidates were obtained from the available transcriptome (Brose et al., 2021). The sequences combining overhangs of *Spe*I and *Not*I restriction sites at the multiple cloning site 1 of the pESC-Leu2d plasmid were synthesized by TwistBioscience (CA, USA) (Ro et al., 2008; Nguyen et al., 2021). The constructs were inserted into pESC already containing the required redox partner cytochrome P450 reductase (CPR) (Ro et al., 2008) by using 5X In-Fusion cloning system (Takara Bio USA Inc.). The yeast strain YPL 150 C:PEP4KO was used for heterologous expression of the CYP candidates following the procedure described before (Nguyen et al., 2022).

### 2.2 Enzyme assays

The *in vivo* CYP activity screening assays were conducted using the established protocol for yeast whole-cell assays in the 96-well plate (Nguyen et al., 2022). Various MIA alkaloid substrates were fed at a final concentration of 10µM into the yeast cultures for 48 h (**Supplementary Figure 13**). The *in vitro* assays were conducted in different buffers: 1 M citrate pH 4, 1 M citrate pH 5, 1 M HEPES pH 6, 1 M HEPES pH 7, 1 M HEPES pH 8, 1 M Tris pH 9, and 1 M Tris pH 10. The *in vitro* reaction condition was performed with 100 µL of 100 µM buffer, 250 µM NADPH, 10 mg total microsomal protein, and 10 µM hirsuteine at 37 °C for 1 h. The yeast cells containing plasmid without CYP construct were used as empty vector controls. The reaction supernatants were collected by centrifugation and filtration with a 0.2 µm syringe filter (Sartorius). The supernatants were injected to ultra-performance liquid chromatography (UPLC) coupled with a Xevo TQ-S Cronos Triple Quadrupole Mass Spectrometer (MS). All UPLC-MS analyses were conducted on an XBridge BEH XP (50 x 2.1 mm, 1.7 μm) column at a flow rate of 0.6 mL.min^–1^. The column was pre-equilibrated in 90% solvent A (water + 0.1% formic acid), and 10% solvent B (acetonitrile + 0.01% formic acid). The eluting conditions were: 0–8 min, 10–50% B; 8.0–8.5 min, 50–100% B; 8.5–9.5 min, 100% B; and 9.5–11 min, 100–10% B to re-equilibrate the column. Immunoblotting experiment of recombinant MsCYP72056 enzyme was conducted as described before (Nguyen et al., 2021). Steady-state enzyme kinetics was conducted by varying the concentration of hirsuteine substrate from 0 to 300 µM in HEPES pH 7.5, at a fixed concentration of NADPH at 250 µM and analyzed using GraphPad Prism 9.4.1 (GraphPad software).

### 2.3 Enzymatic product purification and structural elucidation

To obtain enzymatic products at sufficient yields for structural elucidation, we performed multiple *in vitro* assays of MsCYP72056 containing 10 mL 100 µM HEPES pH 7.5, 250 µM NADPH, 10 mg microsomal protein, and 50 µM hirsuteine at 37 °C for 1 h. Reactions were stopped by adding 1 mL of methanol. The *in vitro* assay supernatants were combined after centrifugation. The crude enzymatic products mixture was extracted from the supernatant by liquid-liquid extraction with chloroform, which was removed *in vacuo* by GeneVac. Concentrated samples were subjected to a Varian semi-preparative HPLC equipped with a Kinetex^®^ 5 μm EVO C18 column (100 Å, 100 × 250 mm) at a flow rate of 1.5 mL.min^–1^. The column was equilibrated in 90% solvent A (water, 0.1% formic acid) and 10% solvent B (acetonitrile, 0.1% formic acid). The eluting conditions were conducted: 0–5 min, 10–20% B; 5–25 min, 20–70% B; 25–27 min, 70–90% B; 27–30 min, 90% B; 30–31 min, 90–10% B; and 31–34 min, 10% B to re-equilibrate the column. Approximately 0.2 mg of each product was dissolved in 600 μL CDCl_3_ and subjected to 1D NMR (^1^H, ^13^C) and 2D NMR (HSQC, HMBC, COSY NOESY) analyses on a Bruker Avance 600 MHz NMR spectrometer. CD analyses were performed with 0.2 mg/mL samples in CH_3_OH on the Jasco J-815 CD spectrophotometer from 200–400 nm.

## 3 Results

### 3.1 Discovery of the first plant spirooxindole synthase

Using OrthoFinder, we generated the orthogroups from the publicly available transcriptomes of ten species, including non-MIA-producing plants, MIA-producing and spirooxindoles-free plants, and spirooxindole-producing plants (see **Materials and method**). We focus on orthogroups containing CYP71 orthologues specific for spiroxindole alkaloid-producing plants such as *M. speciosa* (**Supplementary Figure 2A**) (Bindra, 1973; Ahmad and Salim, 2015; Manwill et al., 2022) as we speculated that member(s) of the CYP71 subfamily could oxidize the seco-/hetero-yohimbine alkaloids to oxygenated intermediates, which would be rearranged to spirooxindole pairs (**Figure 1**). Our analysis identified six candidates, namely MsCYP53813, MsCYP72054, MsCYP72056, MsCYP9580, MsCYP9583, and MsCYP9585 from the orthogroup OG0016157.

**Figure 2:**
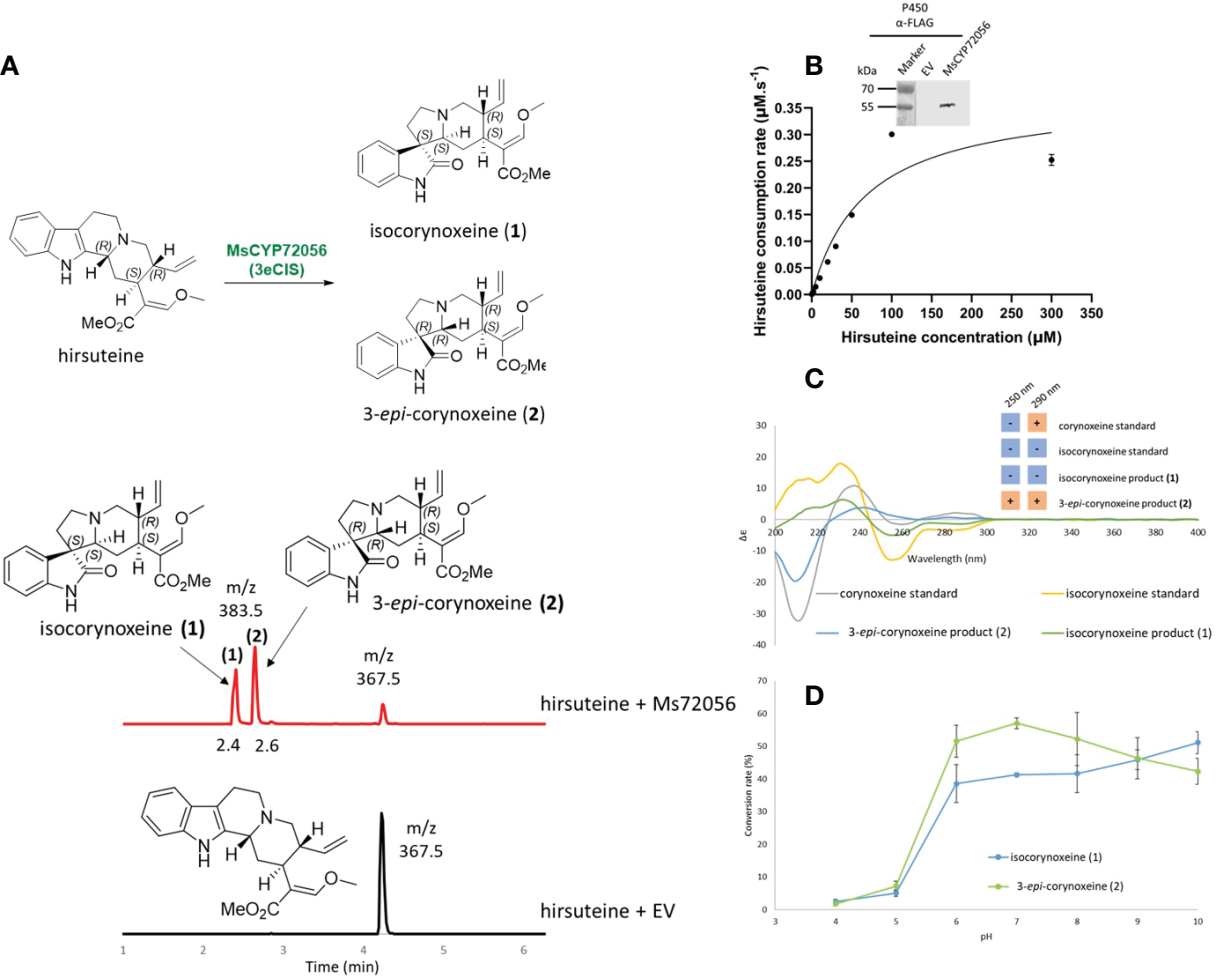
The activity of 3eCIS (MsCYP72056) with hirsuteine as substrate. **(A)** Enzymatic reaction and extracted ion chromatograms from LC-MS analysis showing the *in vitro* activity of MsCYP72056 with hirsuteine in HEPES pH 7.5 buffer. **(B)** Immunoblotting and enzyme kinetics of MsCYP72056 using total microsomal protein extraction of *S. cerevisiae* expressing MsCYP72056/CPR. **(C)** Circular dichroism spectra and Cotton effects at 250 nm and 290 nm of corynoxeine, isocorynoxeine, and enzymatic products **1** and **2**. **(D)** Product profile of *in vitro* assays of recombinant MsCYP72056 at different pHs.

From the spirooxindole structures reported *in M. speciosa*, we traced back to the plausible corynanthe- and ajmalicine-type precursors. Among these, ajmalicine, tetrahydroalstonine, mitragynine, 9-hydroxycorynantheidine, yohimbine, corynanthine, and hirsuteine were available and used for the functional validation of CYP candidates. To test the enzyme activities, 10 μM of the putative substrates were fed to 100-μL YPL154C:PEP4KO yeast cultures for 48 hr. Only yeast cultures harbouring the construct pESC-Leu2d::*CPR*/*MsCYP72056* showed the consumption of hirsuteine ([M+H]^+^ *m/z* 367.5) and the formation of two new products ([M+H]^+^ *m/z* 383.5), **1** at 2.4 min and **2** at 2.6 min, as analyzed by LC-MS/MS (**Supplementary Figure 5A**). A 16-amu difference between the products and the substrate indicated that MsCYP72056 catalyzed an oxygenation/oxidation reaction. No enzymatic product was observed when hirsuteine was incubated with yeast transformed with an empty vector or constructs containing other CYP candidates. *In vitro* assays with microsomal protein of yeast expressing pESC-leu2d::*CPR/MsCYP72056* also showed that in the presence of NADPH, hirsuteine was consumed, resulting in the formation of products **1** and **2** (**Figure 2A**). Michaelis–Menten kinetics characterization of MsCYP72056 with hirsuteine revealed a *K*_M_ value of 68.33 µM. We also investigated the *in vitro* activity of MsCYP72056 in the pH range of 4–10 and found that the enzymatic reaction was more favourable at pH 6–10 (**Figure 2B**). There was a 20-fold increase of isocorynoxeine levels from the same amount of substrate as the pH increased from 4 to 10, of which the most dramatic increase (5% to 50%) was observed as the pH increased from 5 to 6 (**Figure 2D**).

In addition to hirsuteine, 16 other structurally various indole alkaloids were used to assess the substrate scope of MsCYP72056 (**Supplementary Figure 13**). Both *in vivo* and *in vitro* assays using cultures of yeast containing pESC-leu2d::*CPR/MsCYP72056* and its microsomal fractions, respectively, showed that *MsCYP72056* accepted hirsutine beside hirsuteine but not the other alkaloids (**Supplementary Figures 5**, **13**). Similar to hirsuteine, hirsutine is also a (3*R*) secoyohimbine alkaloid with an ethyl group at C-20 in place of a vinyl group in hirsuteine (**Supplementary Figure 5**). LC-MS analysis showed the consumption of hirsutine by MsCYP72056, yielding two products **3** and **4** with 16 amu (*m/z* 385.5) greater than the substrate (*m/z* 369.5). Multiple reaction monitoring (MRM) and daughter scan analyses were performed to detect the oxindole scaffold of the enzymatic products. The specific daughter ion of oxindole ([M+H]^+^ *m/z* 160.0) was observed, confirming that hirsutine was also converted into two spirooxindole products (Avula et al., 2015). Based on MsCYP72056 activity with hirsuteine, we speculated that the products from hirsutine were (3-*epi*-) rhynchophylline-type spirooxindoles, some of which have recently been isolated from *M. speciosa* (Flores-Bocanegra et al., 2020).

### 3.2 Structural elucidation of spirooxindole enzymatic products

To elucidate the stereochemistry of the enzymatic products **1** and **2**, large-scale *in vitro* reactions were conducted. Approximately 0.2 mg of the two products were purified and subjected to 1D NMR (^1^H, ^13^C), 2D NMR (HSQC, HMBC, COSY, NOESY), and circular dichroism (CD) analyses (**Supplementary Figures 7**-**9**). The 1D NMR data confirmed the spirooxindole skeleton of two enzymatic products compared to previously reported compounds corynoxeine and isocorynoxeine (**Supplementary Tables 1**, **2** **;** **Supplementary Figures 7**, **10**) (Kitajiina et al., 2001; Flores-Bocanegra et al., 2020). In particular, the spectra of compound **2** resemble those of corynoxeine, and **1** had identical spectra with isocorynoxeine. In 2D NMR analysis, key NOE signals of isocorynoxeine, such as H-3/H-6, H-5/H-21, and H-20/H-21 were observed in NOESY spectrum of **1** (**Supplementary Table 2** **;** **Supplementary Figures 11B**). These signals initially indicated the 3*S* and 20*R* configurations of C-3 and C-20 in **1** (Seki et al., 1993; Qi et al., 2015). Based on the previous studies in conformational analysis of spirooxindole alkaloids, **1** possessed the *normal*-type conformation as that of mitraphylline and formosanine (Shamma et al., 1967; Seki et al., 1993). As the deshielding effect at H-9 has been reported to differentiate the 7*S* and 7*R* isomers in the *normal*-type spirooxindole alkaloids (Seki et al., 1993), the downfield shift of H-9 in **1** (δ 7.45) depicted the 7*S* configuration (**Supplementary Table 2**). Although **2** had similar 1D NMR data with corynoxeine, we could not observe the key NOE signals of the *normal*-type conformation (3*S*, 20*R*) in the spectra of **2** (**Supplementary Table 1** **;** **Supplementary Figure 8B**). However, we could detect the NOE signals of the *pseudo*-type alkaloids (3*R*, 20*R*) as recently described in two compounds 3-*epi*-rhynchophylline and 3-*epi*-corynoxine B (Flores-Bocanegra et al., 2020). The NOE correlations were among H-3/H-9, H-9/H-6, H-5/H-3, H-18/H-20 (**Supplementary Figure 8B**). Therefore, we proposed that **2** was an epimer of corynoxeine, which was 3-*epi*-corynoxeine (3*R*, 7*R*, 20*R*).

To further confirm our proposed structures, we examined the C-3 and C-7 stereocenters of the enzymatic products of MsCYP72056 and the authentic standards of corynoxeine and isocorynoxeine using circular dichroism (CD) spectroscopic analysis (**Figure 2C**). Intriguingly, the positive Cotton effect at 250 nm confirmed the 3*R* configuration of product **2**. On the contrary, a negative Cotton effect at 250 nm suggested the 3*S* configuration of product **1**. By comparing the CD and NMR spectra of product **1** with isocorynoxeine standard data (**Figure 2C**, **Supplementary Table 2**), we concluded that **1** was isocorynoxeine (3*S*, 7*S*) (**Figure 2A**). Meanwhile, **2** was a 3*R* spirooxindole since it has the opposite Cotton effect at 250 nm, as compared to the corynoxeine standard (**Figure 2C**). Therefore, product **2** (3*R*, 7*R*) was 3-*epi*-corynoxeine (**Figure 2A**). Based on the product profile of the enzymatic reaction, we named this enzyme 3-*epi*-corynoxeine/isocorynoxeine synthase (3eCIS).

## 4 Discussion

Spirooxindole alkaloids have become highly sought-after scaffolds thanks to their stereochemical diversity and substantial range of bioactivities (Nasri et al., 2021). The total synthesis of spiroxindole involves lengthy procedures (up to 17 steps) (Wanner et al., 2013; Zhang et al., 2019), some of which require costly as well as toxic catalysts such as palladium and result in minute yields of products in complex mixtures (Nasri et al., 2021). Although hundreds of tetra- and pentacyclic spirooxindole alkaloids have been discovered (Nasri et al., 2021) and MIA biosynthesis has been studied extensively (Pan et al., 2016; Dang et al., 2017; Dang et al., 2018; Caputi et al., 2018; Farrow et al., 2019; Lopes et al., 2019; Hong et al., 2022; Wang et al., 2022), no enzymes catalyzing the oxidative rearrangement of corynanthe alkaloids to spirooxindole alkaloids have been reported. Our discovery of 3eCIS in *M. speciosa* has provided an answer to the historical question of spirooxindole biosynthesis in plants and highlights the versatility of CYP71 enzymes in MIA scaffolding. From secoyohimbine precursors, 3eCIS catalyzes the formation of polycyclic spirooxindole alkaloids with high yield (10 µg/mL purified product from *in vitro* reaction). In particular, our LC-MS/MS analysis showed a total conversion of hirsuteine substrate to the spirooxindole products at physiological pH and 37°C within 1 hour (**Supplementary Figure 6**). CYP71 enzymes are well known for their roles in MIA metabolism, such as sarpagan bridge enzyme from serpentine wood (*R. serpentina*) (Dang et al., 2018), and geissochizine oxidases from Madagascar periwinkle (*C. roseus*) (Tatsis et al., 2017; Qu et al., 2018) and blackboard tree (*Alstonia scholaris*) (Wang et al., 2022). These notable examples and 3eCIS reported here provide the entry points to various MIA subgroups, including spirooxindole (this work), sarpagan (Dang et al., 2018), strychno (Tatsis et al., 2017; Hong et al., 2022) and akuammilan (Wang et al., 2022) alkaloids. While a flavoprotein monooxygenase was recently reported to catalyze the formation of a spirooxindole alkaloid in fungus, 3eCIS is the first plant enzyme reported to bear the spirooxindole formation catalytic activity (Tsunematsu et al., 2013; Liu et al., 2021). Intriguingly, oxidative rearrangements of the biosynthetic motif of seco-/heteroyohimbine alkaloids have been used to chemically synthesize the spirooxindole core (Xu et al., 2019), and extensive studies in spirooxindole synthesis have been focused on more sustainable synthetic strategies, including less toxic catalysts and less chemical waste (Yan and Wang, 2016; Zhang et al., 2017; Xu et al., 2019). Towards this end, our finding paves the way to access spirooxindole alkaloids and their derivatives through biocatalysis and enzyme engineering.

**Scheme 1:**
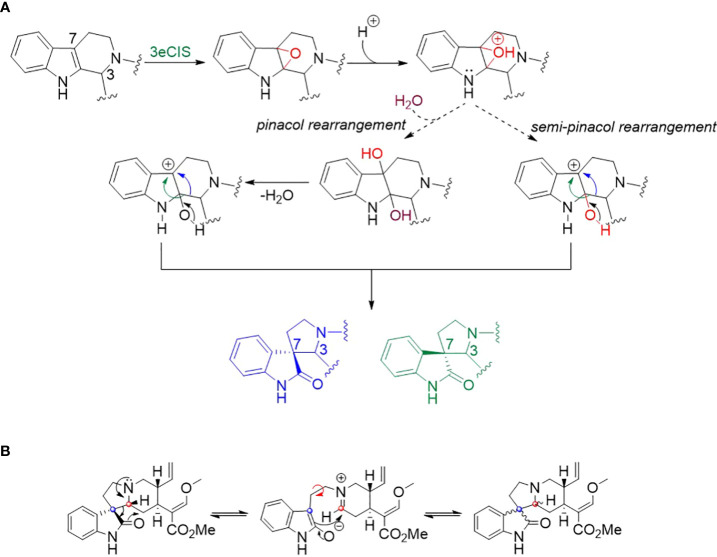
**(A)** Proposed mechanism for the formation of spirooxindole *via* (semi-)pinacol rearrangement. **(B)** The isomerization of spirooxindoles *via* intramolecular Mannich reactions (Laus et al., 1996; Flores-Bocanegra et al., 2020). The red and blue dots correspond to C-3 and C-7, respectively.

Spirooxindoles from kratom are highly diverse in terms of scaffold and stereochemistry (Flores-Bocanegra et al., 2020). The stereogenic centers (C-3, C-7, C-15 and C-20) of spirooxindoles are considered key in deciphering the upstream biosynthetic pathways of spirooxindoles (**Figure 1A**). Previous studies hypothesized that these configurations remain unchanged between an oxindole product and its tetrahydro-β-carboline precursor; therefore, the products **1** and **2** of 3eCIS were initially expected to share the stereo-configurations in rings C and D with those of hirsuteine (3*R*, 20*R*) (**Figure 1B**) (Lopes et al., 2019). Since the *pseudo*-conformation of 3*R* spirooxindoles are not stable due to the interaction of the spirooxindole ring with ring D, the 3*R* spirooxindoles could spontaneously isomerize to the 3*S* spirooxindoles (*normal*-conformation) *via* intramolecular Mannich reaction to reduce the steric hindrance (**Scheme 1B**) (Seki et al., 1993). The C–C single bonds in C-5–C-6–C-7 could freely rotate, resulting in interconvertible 3*R* and 3*S* spirooxindoles through a zwitterion intermediate. In contrast to the configuration retention hypothesis, our newly found enzyme could transform the 3*R* secoyohimbine alkaloid to a mixture of 3*R* and 3*S* spirooxindoles, which can be partially controlled by different pH (**Figure 2D**). In the enzymatic reaction with hirsuteine catalyzed by 3eCIS, in addition to the two characterized products 3-*epi*-corynoxeine and isocorynoxeine, it is possible that other stereoisomers, corynoxeine or 3-*epi*-isocorynoxeine, were generated as we noticed a small peak of *m/z* 383 ([M+H]^+^) in the extracted ion and MRM chromatograms (**Figure 2A**).

Computational and synthetic chemistry studies suggested that a seco-/hetero-yohimbine alkaloid such as hirstuteine can be converted to a pair of spirooxindole epimers by epoxidation on the indole ring followed by (semi-)pinacol rearrangement (**Scheme 1A**) (Xu et al., 2019; Liu et al., 2021). The 3eCIS enzyme is proposed to catalyze the initial oxygenation step (Xu et al., 2019). Then, the formation of carbocation at C-7 could occur without water addition via the (semi-)pinacol mechanism. Subsequently, the ring opening likely allows the alkyl chain at C-3 to rearrange on both sides of the indole ring to yield the spirooxindoles (**Scheme 1A**).

Most spirooxindole alkaloids occur at trace abundance levels *in planta* (Nasri et al., 2021). The discovery and characterization of 3eCIS as reported here provide insights into the long puzzling biosynthesis of plant spirooxindoles and open the gateway to obtaining the elusive spirooxindole alkaloids. The high selectivity towards the (3*R*) tetracyclic corynanthe-type alkaloids of 3eCIS could serve as a starting point for gene discovery and enzyme engineering toward accessing and diversifying spirooxindole core-containing molecules.

## Data availability statement

The original contributions presented in the study are included in the article/**Supplementary Material**. Further inquiries can be directed to the corresponding author.

## Author contributions

T-TTD conceived and designed the project. T-AMN, T-DN, and T-TTD designed the experiments and wrote the manuscript. T-AMN and DG characterized the CYPs *in vitro* and *in vivo*. T-AMN, KC, and ZX characterized the products. All authors contributed to the article and approved the submitted version.
