# Supplementary material for: Discovery of a cytochrome P450 enzyme catalyzing the formation of spirooxindole alkaloid scaffold
Source: Front Plant Sci. 2023 Feb 3;14:1125158. doi: 10.3389/fpls.2023.1125158 (PMC9936145; doi:10.3389/fpls.2023.1125158)
Supplement: Supplementary file 1 [file DataSheet_1.docx]

**Supplementary information**

[**Supplementary Figure 1.** Representative spirooxindole alkaloids isolated from *M. speciosa*. 3](#_Toc121864850)

[**Supplementary Figure 2.** (**A**) Orthogroups analysis of CYP71 enzymes. (**B**) FPKM expression levels of six candidates from OG0016157. 4](#_Toc121864851)

[**Supplementary Figure 3.** Protein alignment of MsCYP candidates. 5](#_Toc121864852)

[**Supplementary Figure 4 .** Phylogenetic tree of MsCYP candidates with other CYPs reported in the biosynthesis of MIAs from different species. 6](#_Toc121864853)

[**Supplementary Figure 5.** Extracted ion chromatograms showing the *in vivo* activity of MsCYP72056 with **(A)** hirsuteine substrate and **(B)** hirsutine substrate. 7](#_Toc121864854)

[**Supplementary Figure 6.**  Chromagram showing the total conversion of hirsuteine substrate into the oxindole products in the *in vitro* assay of freshly collected total microsomal protein Ms72056 in HEPES pH 7.0 buffer. 8](#_Toc121864855)

[**Supplementary Figure 7. (A)**  ^1^H and **(B)** ^13^C NMR spectra of enzymatically produced 3-*epi*-corynoxeine 9](#_Toc121864856)

[**Supplementary Figure 8. (A)** 2D-COSY and **(B)** 2D-NOESY spectra of enzymatically produced 3-*epi*-corynoxeine 10](#_Toc121864857)

[**Supplementary Figure 9. (A)** HSQC and **(B)** HMBC spectra of enzymatically produced 3-*epi*-corynoxeine 11](#_Toc121864858)

[**Supplementary Figure 10. (A)** ^1^H and **(B)** ^13^C NMR spectra of enzymatically produced isocorynoxeine 12](#_Toc121864859)

[**Supplementary Figure 11. (A)** 2D-COSY and **(B)** 2D-NOESY spectra of enzymatically produced isocorynoxeine 13](#_Toc121864860)

[**Supplementary Figure 12. (A)** HSQC and **(B)** HMBC spectra of enzymatically produced isocorynoxeine 14](#_Toc121864861)

[**Supplementary Figure 13.** Substrate specificity of MsCYP72056.. 15](#_Toc121864862)

[**Supplementary Table 1.** ^1^H and ^13^C NMR data of enzymatically produced 3-*epi*-corynoxeine **(2)** (CDCl_3_, 300 K, 600 MHz) 16](#_Toc121864863)

[**Supplementary Table 2.** ^1^H and ^13^C NMR data of enzymatically produced isocorynoxeine **(1)** (CDCl_3_, 300 K, 600 MHz) 18](#_Toc121864864)

[**References** 20](#_Toc121864865)

# **Supplementary Figure 1.** Representative spirooxindole alkaloids isolated from *M. speciosa*.

**
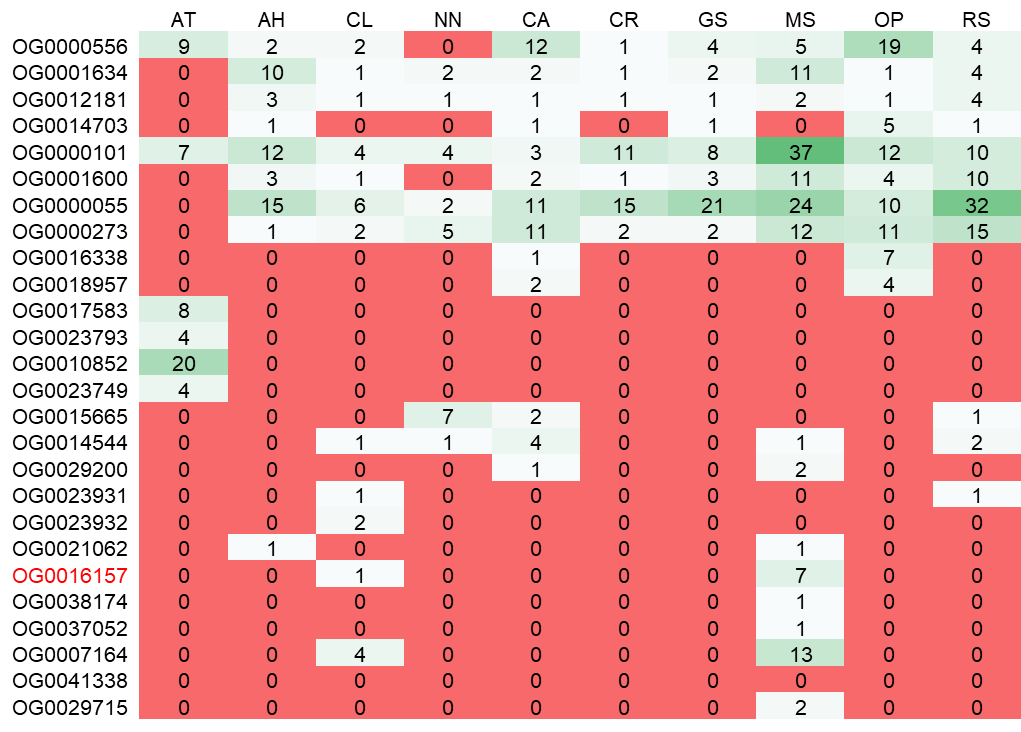
**

**A**

**B**

**
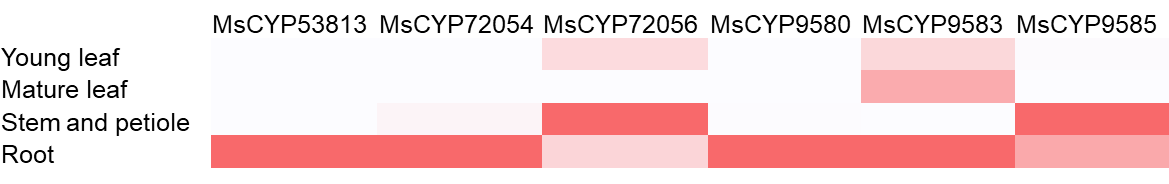
**

# **Supplementary Figure 2.** (**A**) Orthogroups analysis of CYP71 enzymes. (**B**) FPKM expression levels of six candidates from OG0016157 (Brose et al., 2021).

******Supplementary Figure 3.** Protein alignment of MsCYP candidates.

# **Supplementary Figure 4 .** Phylogenetic tree of MsCYP candidates with other CYPs reported in the biosynthesis of MIAs from different species.


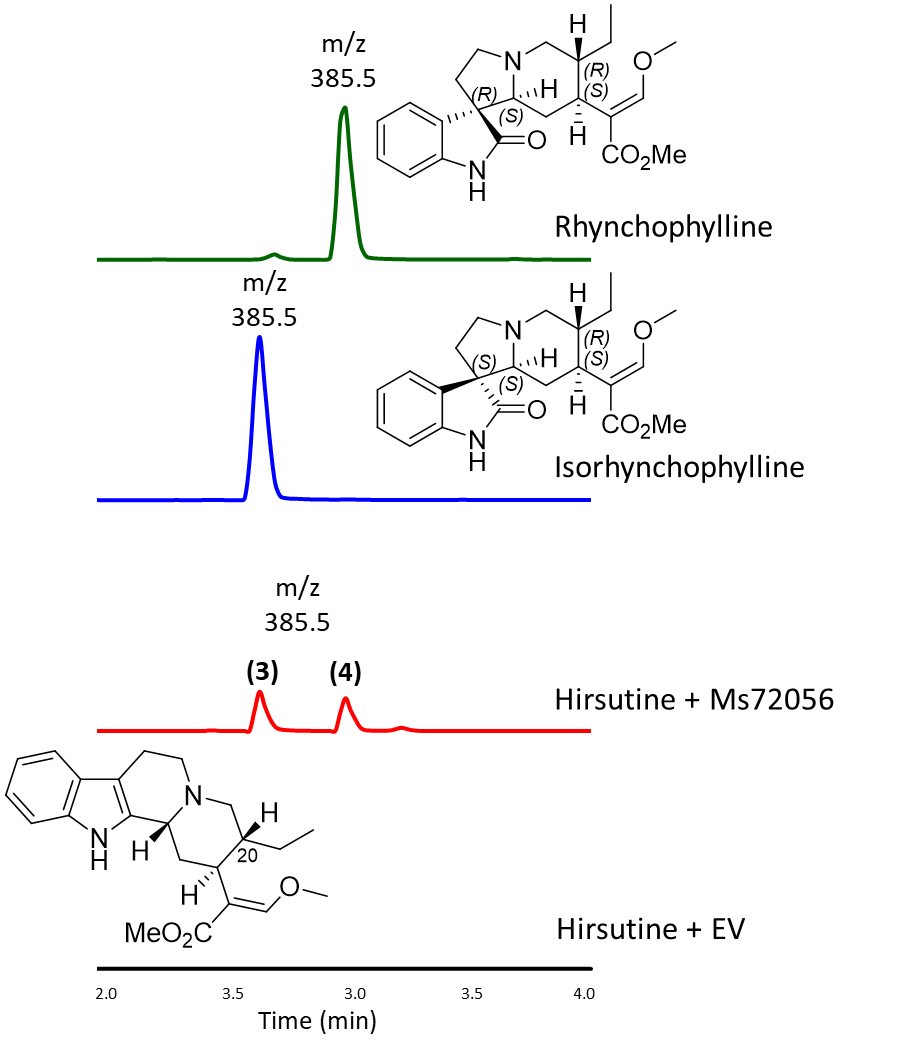
**
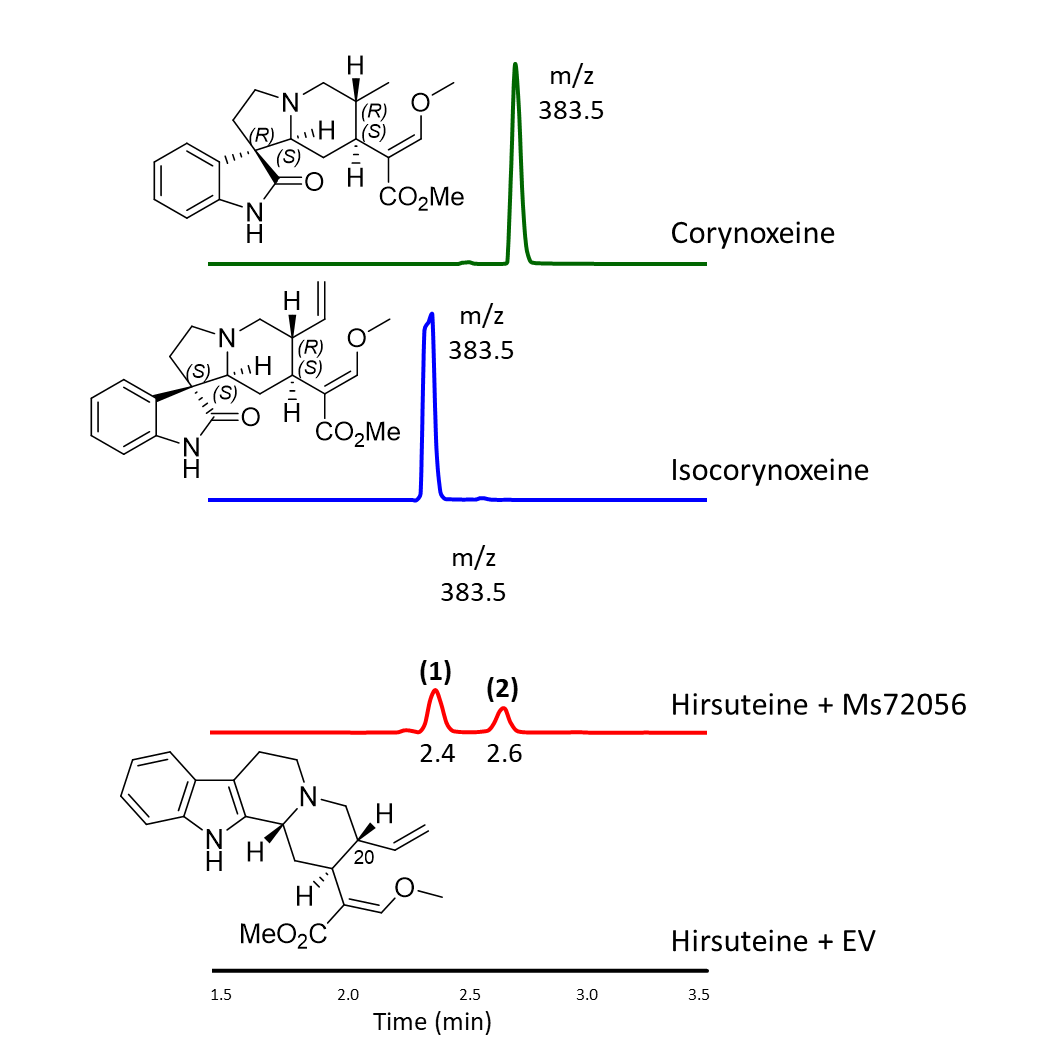
**

**A**

**B**

# **Supplementary Figure 5.** Extracted ion chromatograms showing the *in vivo* activity of MsCYP72056 with **(A)** hirsuteine substrate and **(B)** hirsutine substrate.


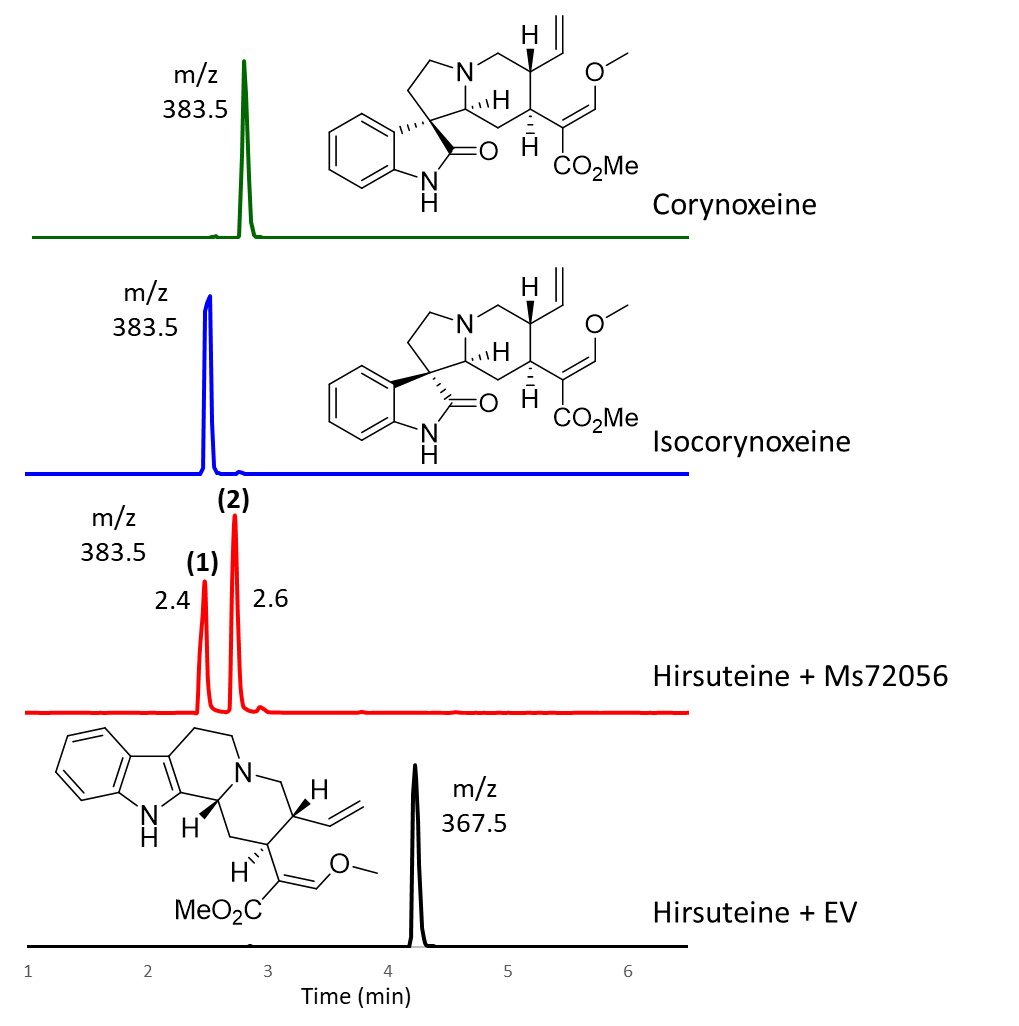


# **Supplementary Figure 6.** Chromatograms showing the total conversion of hirsuteine substrate into the oxindole products in the *in vitro* assay of freshly collected total microsomal protein Ms72056 in HEPES pH 7.0 buffer.


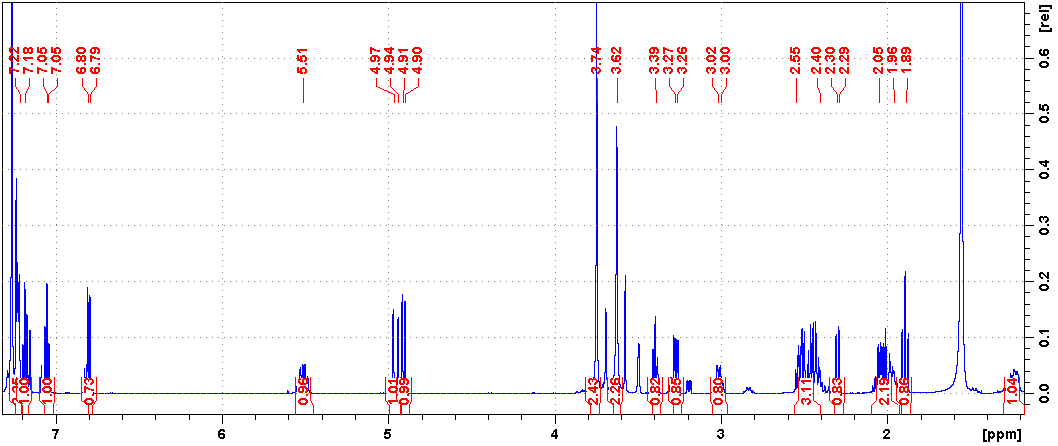
**A**


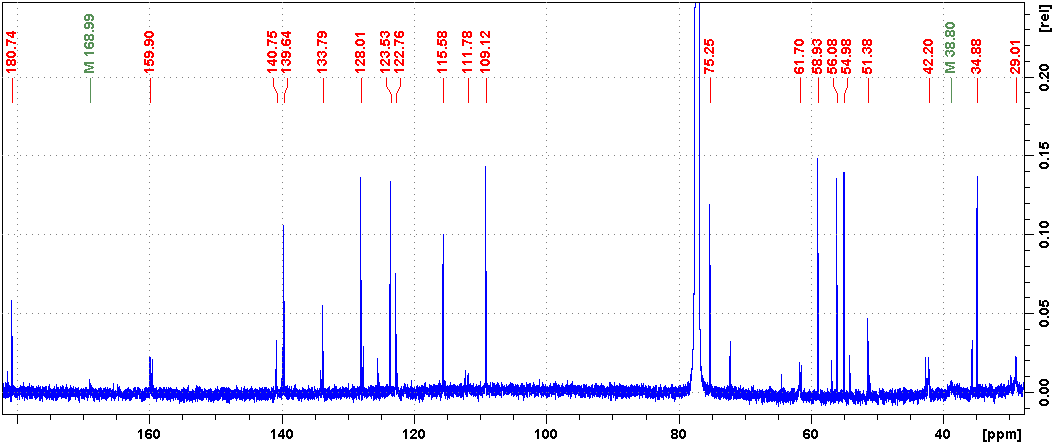
**B**

# **Supplementary Figure 7. (A)** ^1^H and **(B)** ^13^C NMR spectra of enzymatically produced 3-*epi*-corynoxeine


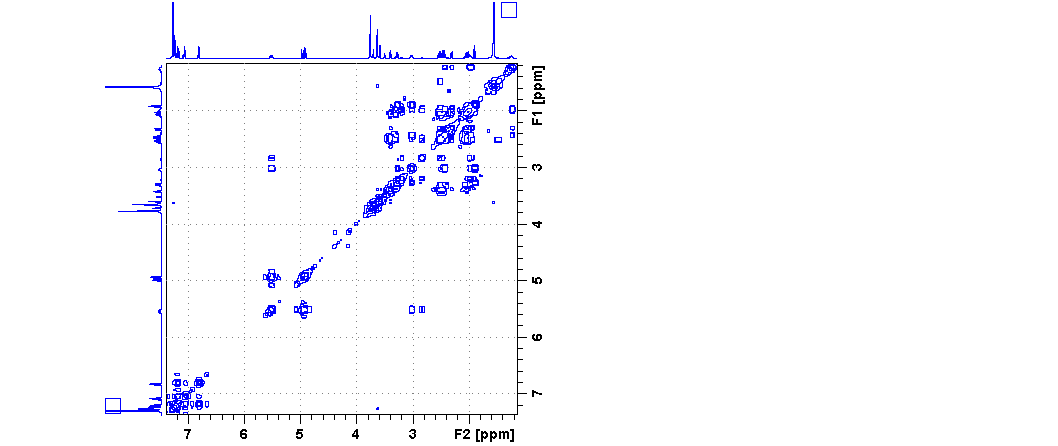
**A**


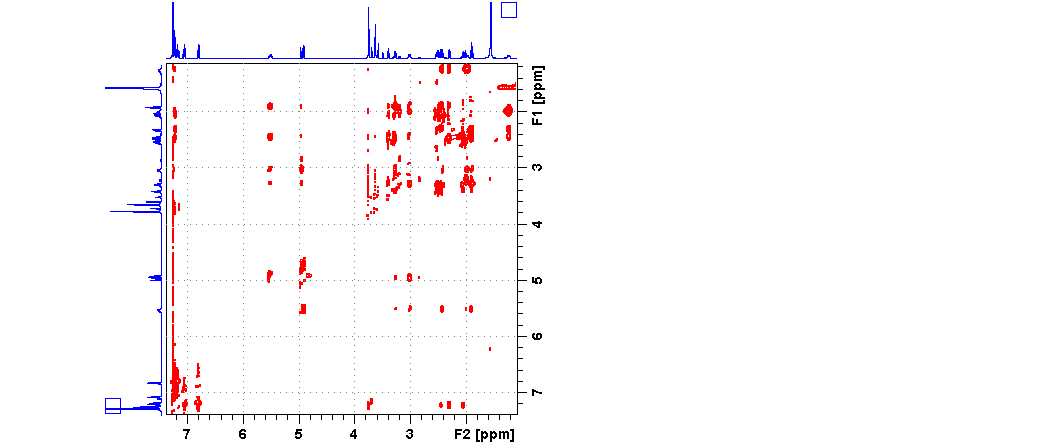
**B**

# **Supplementary Figure 8. (A)** 2D-COSY and **(B)** 2D-NOESY spectra of enzymatically produced 3-*epi*-corynoxeine


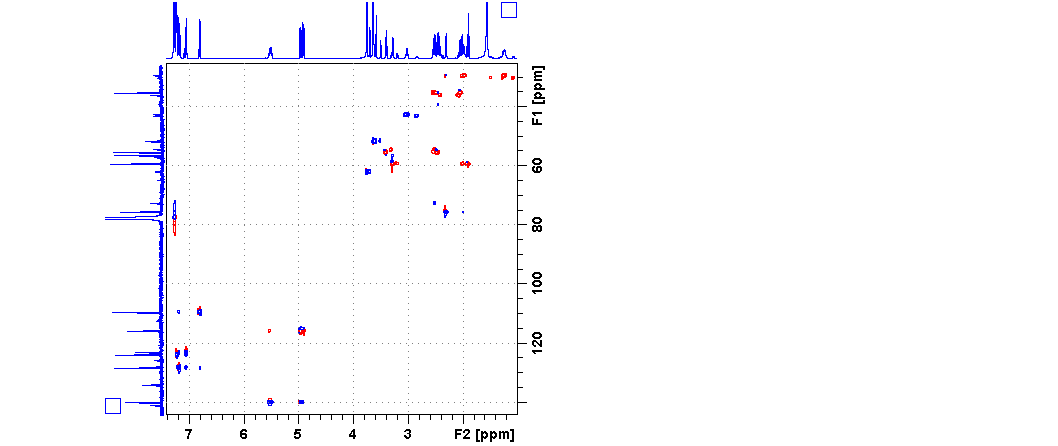
**A**

**B**


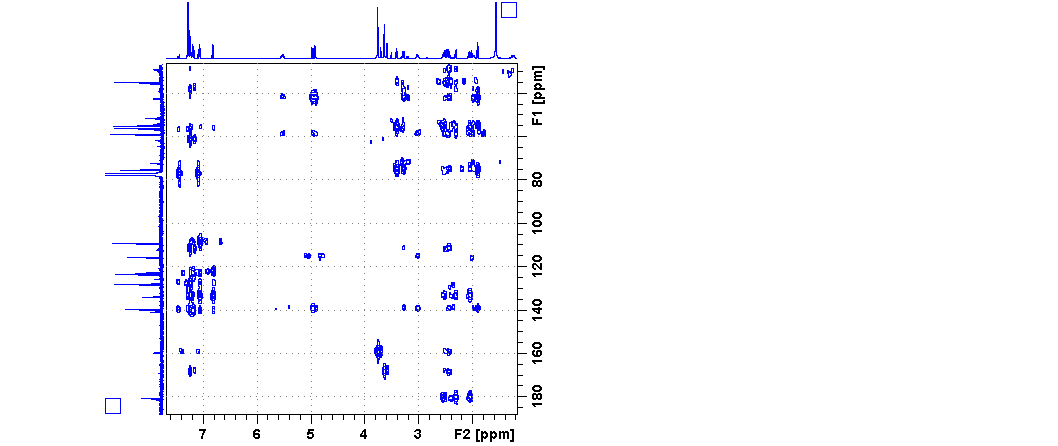


# **Supplementary Figure 9. (A)** HSQC and **(B)** HMBC spectra of enzymatically produced 3-*epi*-corynoxeine


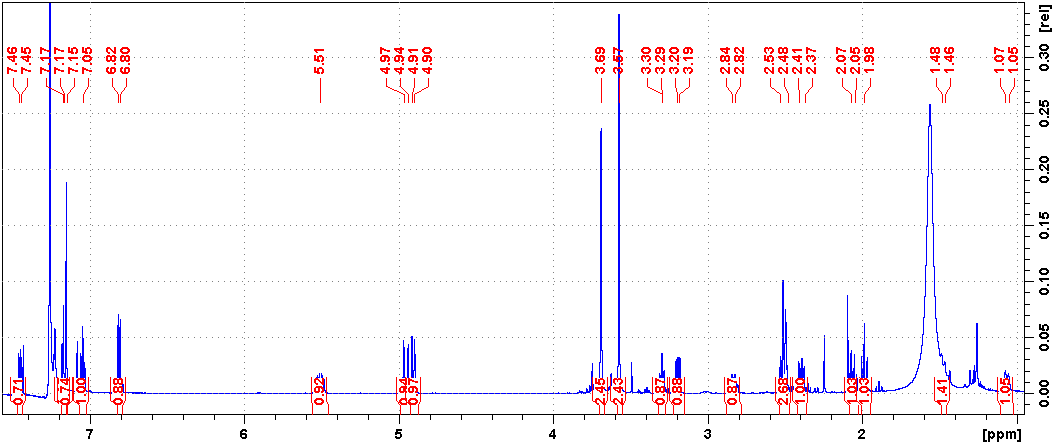
**A**


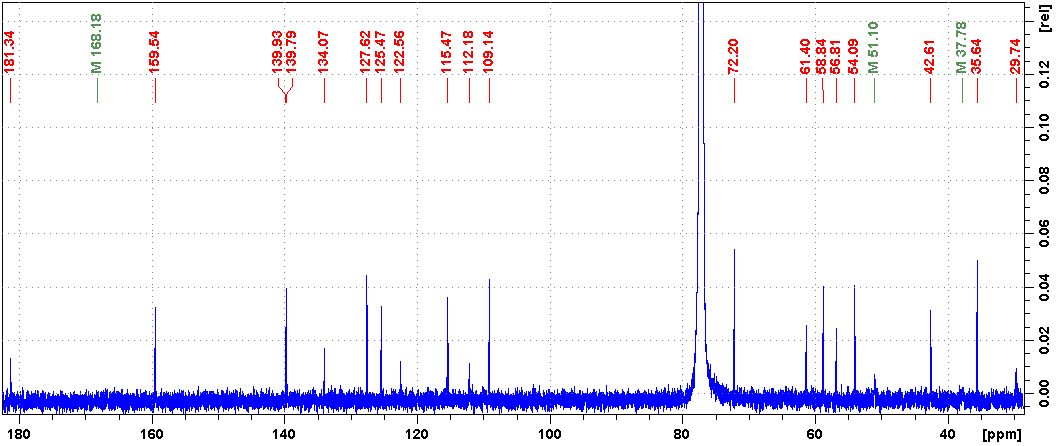
**B**

# **Supplementary Figure 10. (A)** ^1^H and **(B)** ^13^C NMR spectra of enzymatically produced isocorynoxeine


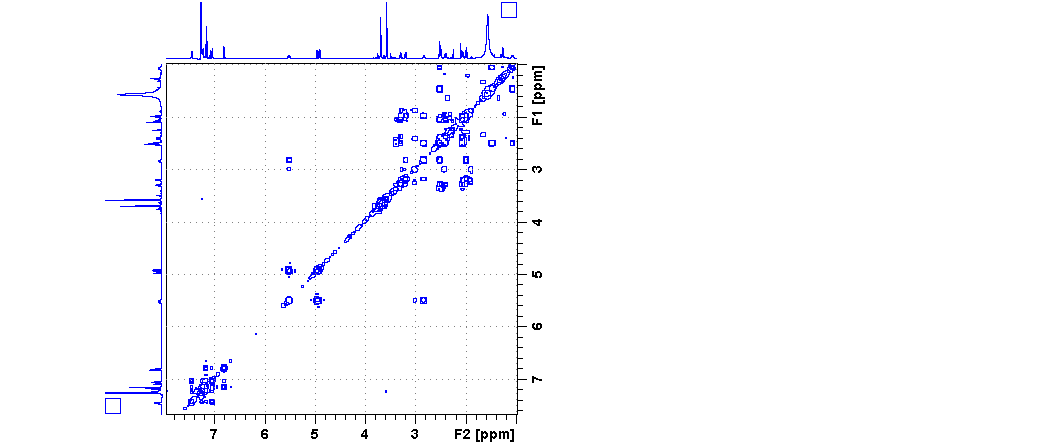
**A**


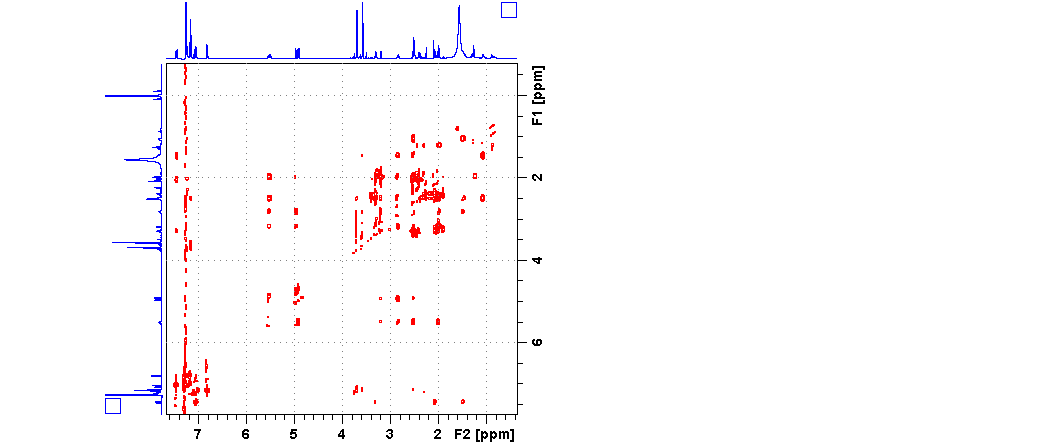
**B**

# **Supplementary Figure 11. (A)** 2D-COSY and **(B)** 2D-NOESY spectra of enzymatically produced isocorynoxeine


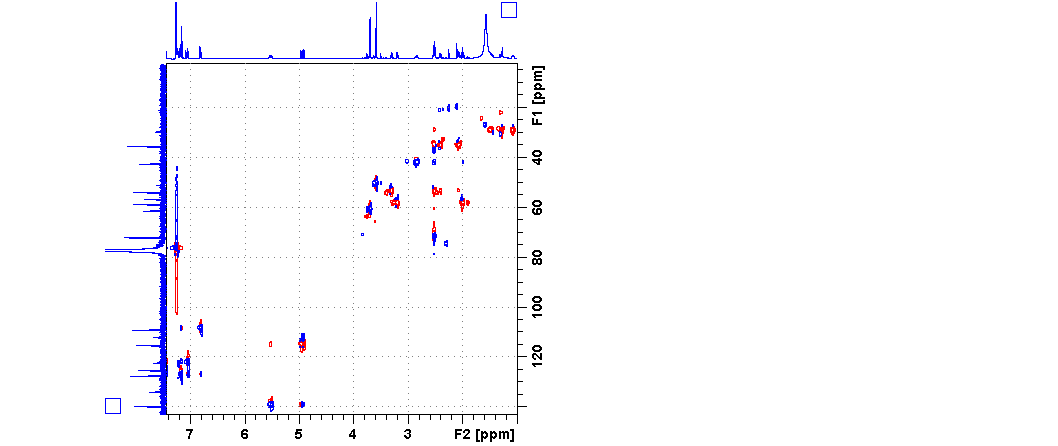
**A**


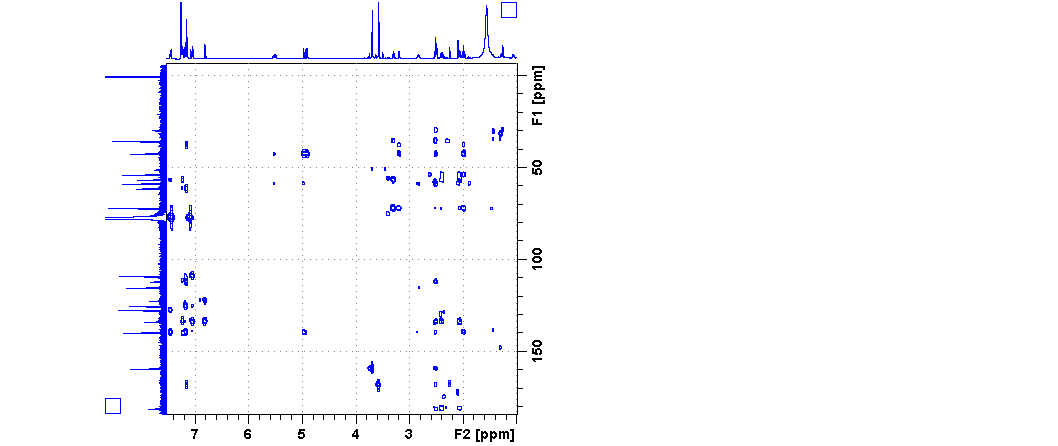
**B**

# **Supplementary Figure 12. (A)** HSQC and **(B)** HMBC spectra of enzymatically produced isocorynoxeine

# **Supplementary Figure 13.** Substrate specificity of MsCYP72056. Only hirsuteine and hirsutine were accepted as substrates at 100 % conversion rate in in vitro assays.

# **Supplementary Table 1.** ^1^H and ^13^C NMR data of enzymatically produced 3-*epi*-corynoxeine **(2)** (CDCl_3_, 300 K, 600 MHz)

| No. | ^1^H | ^1^H(Flores-Bocanegra et al., 2020)  (corynoxeine) | ^13^C | ^13^C(Flores-Bocanegra et al., 2020) (corynoxeine) |
| --- | --- | --- | --- | --- |
| 1 (N**H**) | n/a | 7.53 (1H, s) | n/a | n/a |
| 2 | n/a | n/a | 180.7 | 181.0 |
| 3 | 2.30 (1H, dd, 11.2, 2.5) | 2.30 (1H, dd, 11.3, 2.5) | 75.3 | 75.2 |
| 4 (N) | n/a | n/a | n/a | n/a |
| 5 | 3.39 (1H, t, 8.1)  2.45 (1H, m) | 3.39 (1H, t, 8.3)  2.47 (1H,m) | 55.0 | 55.0 |
| 6 | 2.52 (1H, m)  2.04 (1H, m) | 2.52 (1H, m)  2.04 (1H, ddd, 13.6, 7.2, 1.8) | 34.9 | 34.9 |
| 7 | n/a | n/a | 56.1 | 58.9 |
| 8 | n/a | n/a | 133.8 | 133.8 |
| 9 | 7.22 (1H, d, 7.5) | 7.22 (1H, d, 7.8) | 123.5 | 123.5 |
| 10 | 7.05 (1H, ddd, 7.6, 7.5, 1.1) | 7.05 (1H, td, 7.6, 1.0) | 122.8 | 122.7 |
| 11 | 7.18 (1H, ddd, 7.6, 7.6, 1.1) | 7.18 (1H, td, 7.7, 1.2) | 128.0 | 128.0 |
| 12 | 6.80 (1H, d, 7.6) | 6.82 (1H, d, 7.7) | 109.1 | 109.2 |
| 13 | n/a | n/a | 140.8 | 140.8 |
| 14 | 2.46 (1H, m)  1.89 (1H, t, 10.8) | 2.47 (1H, m)  1.24 (1H, t, 10.9) | 29.0 | 28.9 |
| 15 | 3.01 (1H, qd, 12.0, 4.2) | 3.01 (1H, qd, 11.5, 3.8) | 38.8 | 38.4 |
| 16 | n/a | n/a | 111.8 | 111.0 |
| 17 | 7.23 (1H, s) | 7.24 (1H, s) | 159.9 | 159.9 |
| 18 | 4.96 (1H, ddd, 17.2, 2.0, 0.8)  4.91 (1H, dd, 10.3, 2.0) | 4.95 (1H, ddd, 17.2, 2.01. 0.8)  4.90 (1H, dd, 10.2, 2.1) | 115.6 | 115.6 |
| 19 | 5.51 (1H, m) | 5.51 (1H, dt, 18.0, 9.1) | 139.6 | 139.6 |
| 20 | 1.97 (1H, dd, 11.0, 4.1) | 1.97 (1H, dd, 12.8, 10.3) | 42.2 | 42.2 |
| 21 | 3.27 (1H, dd, 10.9, 4.0)  1.89 (1H, t, 10.9) | 3.27 (dd, 10.8, 4.1) | 58.9 | 56.1 |
| **C**O_2_Me | n/a | n/a | 169.0 | 169.7 |
| CO_2_**Me** | 3.62 (3H, s) | 3.62 (3H, s) | 51.4 | 51.4 |
| O**Me** | 3.74 (3H, s) | 3.74 (3H, s) | 61.7 | 61.7 |

# **Supplementary Table 2.** ^1^H and ^13^C NMR data of enzymatically produced isocorynoxeine **(1)** (CDCl_3_, 300 K, 600 MHz)

| No. | ^1^H | ^1^H(Kitajiina et al., 2001) | ^13^C | ^13^C(Kitajiina et al., 2001) |
| --- | --- | --- | --- | --- |
| 1 (N**H**) | can’t detect | 7.30 (1H, s) | n/a |  |
| 2 | n/a | n/a | 181.3 | 181.3 |
| 3 | 2.50 (1H, m) | 2.50 (1H, m) | 72.2 | 72.0 |
| 4 (N) | n/a | n/a | n/a |  |
| 5 | 3.30 (1H, ddd, 8.7, 8.7, 2.2)  2.50 (1H, m) | 3.30 (1H, ddd, 8.5, 8.5, 2.2)  2.50 (1H, m) | 54.1 | 53.9 |
| 6 | 2.06 (1H, m)  2.39 (1H, ddd, 13.4, 9.2, 2.2) | 2.08 (1H, ddd, 12.8, 8.5, 4.3)  2.39 (1H, m) | 35.6 | 35.5 |
| 7 | n/a | n/a | 56.8 | 56.6 |
| 8 | n/a | n/a | 134.1 | 133.9 |
| 9 | 7.45 (1H, d, 7.5) | 7.45 (1H, br-d, 7.6) | 125.5 | 125.3 |
| 10 | 7.05 (1H, ddd, 7.5, 7.5, 0.8) | 7.05 (1H, ddd, 7.8, 7.6, 1.0) | 122.6 | 122.4 |
| 11 | 7.17 (1H, ddd, 7.7, 7.7, 1.3) | 7.17 (1H, ddd, 7.8, 7.6, 1.5) | 127.6 | 127.5 |
| 12 | 6.81 (1H, d, 7.7) | 6.80 (1H, d, 7.8) | 109.1 | 109.0 |
| 13 | n/a | n/a | 139.9 | 139.8 |
| 14 | 1.06 (1H, d, 12.3)  1.47 (1H, 12.3) | 1.06 (1H, m)  1.47 (1H, d, m) | 29.7 | 29.6 |
| 15 | 2.51 (1H, m) | 2.50 (1H, m) | 37.8 | 38 |
| 16 | n/a | n/a | 112.2 | 112.0 |
| 17 | 7.15 (1H, s) | 7.15 (1H, s) | 159.5 | 159.4 |
| 18 | 4.91 (1H, dd, 10.2, 1.9)  4.95 (1H, dd, 17.2, 1.9) | 4.90 (1H, dd, 10.3, 2.2)  4.95 (1H, dd, 17.4, 2.2) | 115.5 | 115.3 |
| 19 | 5.51 (1H, m) | 5.50 (1H, ddd, 17.4, 10.3, 8.0) | 139.8 | 139.6 |
| 20 | 2.83 (1H, m) | 2.83 (1H, m) | 42.6 | 42.4 |
| 21 | 1.99 (1H, t, 11.0)  3.19 (1H, dd, 11.0, 4.1) | 1.98 (1H, dd, 10.7, 10.7)  3.19 (1H, d, 10.7, 3.9) | 58.8 | 58.7 |
| **C**O_2_Me | n/a | n/a | 168.2 | 168 |
| CO_2_**Me** | 3.57 (3H, s) | 3.58 (3H, s) | 51.1 | 50.9 |
| O**Me** | 3.69 (3H, s) | 3.69 (3H, s) | 61.4 | 61.3 |

# **References**

Brose, J., Lau, K. H., Dang, T. T. T., Hamilton, J. P., Martins, L. D. V., Hamberger, B., et al. (2021). The Mitragyna speciosa (Kratom) Genome: A resource for data-mining potent pharmaceuticals that impact human health. *G3: Genes, Genomes, Genetics* 11. doi: 10.1093/g3journal/jkab058.

Flores-Bocanegra, L., Raja, H. A., Graf, T. N., Augustinović, M., Wallace, E. D., Hematian, S., et al. (2020). The Chemistry of Kratom [ Mitragyna speciosa]: Updated Characterization Data and Methods to Elucidate Indole and Oxindole Alkaloids. *J Nat Prod* 83, 2165–2177. doi: 10.1021/acs.jnatprod.0c00257.

Kitajiina, M., Yokoya, M., Takayama, H., and Aimi, N. (2001). Co-occurrence of Harman and β-Carboline-type Monoterpenoid Glucoindole Alkaloids in Ufia de Gato (Uncaria tomentosa). *Natural Medicines* 55, 308–310.
